# Facial Neuralgia the Result of Chronic Rheumatism

**Published:** 1861-02

**Authors:** 


Facial Neuralgia the Result of Chronic Rheumatism.—This patient some of you have seen before, when walking the wards with me. (See *Reporter* of September 1st.) He came here on the 18th of last month, is a seaman, and had been ailing for some time with chronic subacute rheumatism, stiffness of his joints, and difficulty of motion. But what attracted our attention more particularly was, that he could not close his right eye, could not move with ease the right side of his mouth, could not whistle, that is to say, he lacked the power of co-ordinating the muscular motions necessary for that performance. He had no pain.

In cases of this kind it is of the utmost importance to determine upon what causes this loss of power depends. It may be owing, as in this case, to some rheumatic affection, perhaps thickening of the sheath of the nerve; then it is simple local paralysis; or it may depend upon cerebral lesions. In regard to prognosis as well as treatment, a correct diagnosis is necessary.

Now, this patient has no headache, he sleeps well, he has perfect control over his tongue, showing that the hypoglossal nerve is not affected, the orbicularis and levator palpebræ on that side act well; in short, the absence of any symptoms, except the local loss of power, sufficiently marks the case as one not very grave. The treatment has been anti-rheumatic; iodide of potassium, blistering behind the ear, and a pill two or three times a day, composed of half a grain of extract of nux vomica, two grains of sulphate of quinine, and the same quantity of powdered alum.—*Medical and Surgical Reporter.*

